# *In silico* and *in vitro* arboviral MHC class I-restricted-epitope signatures reveal immunodominance and poor overlapping patterns

**DOI:** 10.3389/fimmu.2022.1035515

**Published:** 2022-11-17

**Authors:** Ágata Lopes-Ribeiro, Franklin Pereira Araujo, Patrícia de Melo Oliveira, Lorena de Almeida Teixeira, Geovane Marques Ferreira, Alice Aparecida Lourenço, Laura Cardoso Corrêa Dias, Caio Wilker Teixeira, Henrique Morais Retes, Élisson Nogueira Lopes, Alice Freitas Versiani, Edel Figueiredo Barbosa-Stancioli, Flávio Guimarães da Fonseca, Olindo Assis Martins-Filho, Moriya Tsuji, Vanessa Peruhype-Magalhães, Jordana Grazziela Alves Coelho-dos-Reis

**Affiliations:** ^1^ Laboratório de Virologia Básica e Aplicada, Instituto de Ciências Biológicas, Departamento de Microbiologia, Universidade Federal de Minas Gerais, Belo Horizonte, Brazil; ^2^ Grupo Integrado de Pesquisas em Biomarcadores, Instituto René Rachou, Fundação Oswaldo Cruz, Belo Horizonte, Brazil; ^3^ Laboratorio de Genética Celular e Molecular, Instituto de Ciências Biológicas, Departamento de Genética, Universidade Federal de Minas Gerais, Belo Horizonte, Brazil; ^4^ Department of Pathology da University of Texas Medical Branch, Galveston, TX, United States; ^5^ Centro de Tecnologia em Vacinas da UFMG, Universidade Federal de Minas Gerais, Belo Horizonte, Brazil; ^6^ Aaron Diamond AIDS Research Center, Irving Medical School, Columbia University, New York City, NY, United States

**Keywords:** MHC-I peptides, CD8^+^T cell response, arbovirus, overlapping peptides, HLA-A2-restricted peptides, immunoinformatics

## Abstract

**Introduction:**

The present work sought to identify MHC-I-restricted peptide signatures for arbovirus using in silico and in vitro peptide microarray tools.

**Methods:**

First, an in-silico analysis of immunogenic epitopes restricted to four of the most prevalent human MHC class-I was performed by identification of MHC affinity score. For that, more than 10,000 peptide sequences from 5 Arbovirus and 8 different viral serotypes, namely Zika (ZIKV), Dengue (DENV serotypes 1-4), Chikungunya (CHIKV), Mayaro (MAYV) and Oropouche (OROV) viruses, in addition to YFV were analyzed. Haplotype HLA-A*02.01 was the dominant human MHC for all arboviruses. Over one thousand HLA-A2 immunogenic peptides were employed to build a comprehensive identity matrix. Intending to assess HLAA*02:01 reactivity of peptides in vitro, a peptide microarray was designed and generated using a dimeric protein containing HLA-A*02:01.

**Results:**

The comprehensive identity matrix allowed the identification of only three overlapping peptides between two or more flavivirus sequences, suggesting poor overlapping of virus-specific immunogenic peptides amongst arborviruses. Global analysis of the fluorescence intensity for peptide-HLA-A*02:01 binding indicated a dose-dependent effect in the array. Considering all assessed arboviruses, the number of DENV-derived peptides with HLA-A*02:01 reactivity was the highest. Furthermore, a lower number of YFV-17DD overlapping peptides presented reactivity when compared to non-overlapping peptides. In addition, the assessment of HLA-A*02:01-reactive peptides across virus polyproteins highlighted non-structural proteins as “hot-spots”. Data analysis supported these findings showing the presence of major hydrophobic sites in the final segment of non-structural protein 1 throughout 2a (Ns2a) and in nonstructural proteins 2b (Ns2b), 4a (Ns4a) and 4b (Ns4b).

**Discussion:**

To our knowledge, these results provide the most comprehensive and detailed snapshot of the immunodominant peptide signature for arbovirus with MHC-class I restriction, which may bring insight into the design of future virus-specific vaccines to arboviruses and for vaccination protocols in highly endemic areas.

## 1 Introduction

Arthropod-borne viruses (arboviruses) comprise a group of highly efficient viruses transmitted by mosquito bite, which are of huge concern for public health authorities. Amongst these viruses are Yellow Fever (YFV), Zika (ZIKV), Dengue (DENV), Chikungunya (CHIKV), Mayaro (MAYV) and Oropouche (OROV). These viruses induce several similar clinical symptoms such as fever, headache, chills, pruritus, exanthema, arthralgia, myalgia and retro-orbital pain (1, 2). No direct-acting antivirals are available for treating these viral infections and mosquito control strategies have been arduous to implement in endemic regions.

As cases of new infections for those arboviruses are constant for seasonal periods in endemic tropical areas, differential diagnosis and clinical management are usually a challenge. Only two of these arboviruses, YFV and DENV, present vaccines approved for public use. Yellow fever vaccines (17D and 17DD) are widely used and are the main prophylactic measure to halt the disease. Regarding the licenced DENV polyvalent vaccine Dengvaxia^®^ (Sanofi Pasteur), this vaccine is designed to prevent Dengue for all the four serotypes, but it still shows restrictions and only seropositive individuals can be vaccinated in endemic areas (3). This vaccine increases the risk of severe dengue in those who experience their first natural dengue infection after vaccination (seronegative individuals) (3).

In line with the scarce resources to countermeasure arboviral infections worldwide, it is yet to be know how protective immunity is established during a primary viral infection or during vaccination. Several correlates of protection have been reported for such viral infections, including neutralizing antibodies, assessed by plate reducing neutralizing antibody (PRNT) tests as well as CD8^+^ T cell responses, quantified by immunological cellular assays (3–7). Among those two, protection is usually associated to the seroconversion, which corresponds to the development of high PRNT levels post vaccination and primary infection (8, 9).

In this regard, while neutralizing antibodies are usually highly specific to 10-25 amino acid (aa) conformational epitopes unique to each viral protein, cross-reactivity in serologic tests still exists amongst patients that had any history of flavivirus infection (10–12). However, despite being described for non-neutralizing antibodies, several studies have also shown that antibody-dependent enhancement effect can be observed for neutralizing antibodies, indicating that the described cross-reactivity for flavivirus may not be associated to protection but rather to problems such as disease severity and difficulty in the differential diagnosis of such infections (11–14). In the context of cellular responses, CD8^+^ T cells are directed to small, linear 9–11 viral sequences that are capable of binding to a nascent MHC-I molecule (15–18) with a possibility of overlap. Therefore, considering the viral genomic similarities amongst this arbitrary group of viruses and the intersecting areas, which are endemic and epidemic to them, there is a possible coincidence in patterns of CD8^+^ T cell recognition amongst arboviruses, that deserves detailed scrutiny.

In the context of profiling immunodominant epitopes of arbovirus, most reports target MHC-class II and B cell epitopes, which are associated to antibody responses (19). In these assays, serum samples from patients are employed to define peptide signatures significant to antibody binding. In terms of peptides with MHC-class I restriction, technical restrictions impair high throughput assays for epitope mapping employing peptides that may activate CD8^+^ T cells (20). The need of fresh antigen presenting cells as well as autologous CD8^+^ T cells from several donors are still an obstacle for broad epitope immunogenicity mapping.

Therefore, in the present study, we sought to identify peptide signatures for arbovirus associated to inducing CD8^+^ T-cell responses using *in-silico* and *in vitro* peptide microarray tools. First, an *in-silico* analysis of immunogenic epitopes restricted to four human MHC class-I most prevalent worldwide was performed followed by the assessment of HLA-A*02:01 reactivity of peptides *in vitro*, using a peptide microarray designed and generated using a dimeric protein containing HLA-A*02:01. Together, the present study provides a snapshot of immunodominant peptide signature for arbovirus with MHC-class I restriction, which may bring insight into the design of future virus-specific vaccines to arboviruses and also for vaccination protocols in highly endemic areas.

## 2 Material and methods

### 2.1 Dataset collection

Polyprotein sequences of the Yellow Fever 17DD vaccine virus (YFV-17DD, Genbank: U17066.1 and DQ100292.1), wild strains of Yellow Fever virus (YFV Asibi, Genbank: AWG42197.1; YFV Outbreak, Genbank: MF538784.2, MF370533.1 and MF405338.1; YFV Pre-Outbreak, Genbank: JF912190.1, JF912188.1), Zika virus (ZIKV, Genbank: KU926310.2, MF352141.1, KX197205.1 and KU527068.1), Dengue virus serotypes 1-4 (DENV1-4, Genbank: JX669466. 1, KP188543.1, KP188551.1, JX286516.1, JX669477.1, KP188556.1, GU131865.1, GU131877.1, GU131844.1 and KJ596662.1), Chikungunya virus (CHIKV, Genbank: KY704952.1, KY704954.1 and KY704947.1), Mayaro virus (MAYV, Genbank: KM400591.1, KY618136.1, KT818520.1, KY618131.1 and KY618132.1) and Oropouche virus (OROV, Genbank: KP795103.1, KP795100. 1, KP795088.1, MG747607.1, AF484424.1, AWU67042.1, AWU67043.1) were acquired on the NCBI Nucleotide database (https://www.ncbi.nlm.nih.gov/nucleotide/).

All collected sequences were used as input to NetCTL 1.2 web software (http://www.cbs.dtu.dk/services/NetCTL/) for prediction of peptides with restriction to HLA-A1, HLA-A2, HLA-A3 or HLA-A24. Peptide prediction was based on MHC-I affinity, peptide cleavage and transport by transporter associated with antigen protein complex (TAP) (21). Only 9-mer peptides with Combined Score values above to 0.75 were considered immunogenic for CD8^+^T cells and maintained for further analysis. This evaluation encompassed four of the most prevalent MHC-I haplotypes in Brazilian population (22).

### 2.2 *In vitro* assessment of HLA-A*02-restricted peptide repertoire using peptide microarray

Evaluation of the HLA-A*02-restricted immunodominant peptide repertoire was performed using PEPperCHIP^©^ (PEPperPRINT^©^, Heidelberg, Germany) custom peptide microarray (**Supplementary Figure 1**). All predicted peptides for YFV-17DD virus, YFV wild strains, DENV1-4, ZIKV, CHIKV, MAYV and OROV with combined score values above 0.75 were included in this microarray, including non-overlapping peptides. **Supplementary Figure 1** shows a schematic compendium for the detection of HLA-A2-peptide binding using PEPperCHIP^©^ microarray (PEPperPRINT^©^, Heidelberg, Germany).

For the microarray, 1,350 peptides were adsorbed in duplicate on the inside of spots located on a microarray glass slide (75.4 mm x 25.0 mm x 1 mm). The first step of the assay consisted of pre-labeling the microarray glass slide with anti-murine IgG1/Cy3 secondary antibody (BD Biosciences, California, USA), for exclusion of background reactivity. For this, the microarray slide was incubated with standard buffer [phosphate-saline buffer (PBS) + 0.05% Tween20; pH 7.4] for 15 minutes at room temperature. After complete removal of the buffer, a new incubation was performed with blocking buffer [PBS + 0.05% Tween20 + 1% bovine serum albumin (BSA); pH 7.4] for 30 minutes at room temperature. After complete removal of the buffer, the microarray slide was incubated with anti-murine IgG1/Cy3 secondary antibody (BD Biosciences, San Jose, CA, USA) previously diluted (1:5,000) in staining buffer [‘standard buffer ‘+ 10%’ blocking buffer ‘]. Incubation was maintained for 45 minutes, at room temperature and protected from light. After complete removal of the secondary antibody, the microarray slide was washed three times in standard buffer, air-dried and digitized.

After the background staining, the microarray slide was incubated with standard buffer at room temperature for 15 minutes. After complete removal of the buffer, 1µg/mL, 10µg/mL and 30µg/mL of HLA-A*02:01 dimer (HLA-A2:β2M:Ig Protein) (23) diluted in staining buffer were added to the microarray glass slide and incubated for 16 hours at 2-8°C. After complete removal of the HLA-A*02:01 dimer, the microarray slide was washed three times using standard buffer and incubated with anti-murine IgG1/Cy3 secondary antibody (BD Biosciences, San Jose, CA, USA) previously diluted (1:5,000) in staining buffer. Incubation was maintained for 45 minutes, at room temperature and protected from light. After complete removal of the secondary antibody, the microarray slide was washed three times in standard buffer, air-dried and digitized.

Throughout the assay, all incubations were performed under constant agitation in an orbital agitation system (140 rpm). The PEPperCHIP^©^ microarray slide (PEPperPRINT^©^, Heidelberg, Germany) was digitized on the Affymetrix 428 Array Scanner device (Thermo Fisher, California, USA). Data regarding HLA-A2:β2M:Ig reactivity to adsorbed peptides was pre-analyzed using PepSlide^®^ Analyzer (PEPperPRINT ^©^, Heidelberg, Germany) and quantified in 16 bit grey-scale images. Results were expressed in fluorescence intensity.

#### 2.2.1 Identification of peptides origin within complete arbovirus polyprotein

Peptide adsorption on the microarray glass slide followed an arbitrary order, therefore it was necessary to assess the origin of reactive peptides within viral polyprotein. For this, all peptides derived from the 17DD vaccine virus and from wild strains of YFV, DENV1-4, ZIKV, CHIKV, MAYV and OROV underwent multiple alignment with the complete polyprotein sequence of the respective virus using MUSCLE alignment algorithm (24) in the software Unipro UGENE v.26 (25). To guarantee a complete evaluation of all arboviruses, peptides shared between two or more viruses were kept in replicate.

### 2.3 *In silico* evaluation of arbovirus derived peptides with YFV-17DD overlapping

#### 2.3.1 Identification of overlapping peptides

Peptides with complete or partial overlapping to YFV-17DD peptides were identified using an identity matrix generated in Geneious 5.5 software (https://www.geneious.com) after multiple alignment applying MUSCLE alignment algorithm (24, 26, 27) in the same software. Shared peptides between all assessed arbovirus were identified and used to assemble a Venn diagram (http://bioinformatics.psb.ugent.be/webtools/Venn/). The results obtained for overlapping peptides between YFV-17DD virus and ZIKV, DENV1-4, CHIKV, MAYV and OROV were displayed in heatmap Additional alignments were performed using MUSCLE algorithm on Unipro UGENE v.33 software.

#### 2.3.2 YFV-17DD polyprotein hydrophobicity and assessment of conserved residues in peptides with different HLA-A*02 affinity ranges

Data regarding the hydrophobicity of complete arboviruses polyprotein was acquired with ProtScale software (https://web.expasy.org/protscale/), using Kyte & Doolittle scale (28). For the assessment of conserved residues in association with peptide immunodominance, global median was stablished for peptide-HLA-A*02 binding affinity obtained *in silico*. The global median values allowed the identification of sequences with high, medium and low values for all three parameters. Two sequences for each virus in all three value ranges were selected for multiple alignment, performed with MUSCLE alignment algorithm (24) in Geneious 5.5 software (26, 29, 30) (**Supplementary Figure 3**). Evaluation of amino acid frequency of aligned sequences was performed using Molecular Evolutionary Genetics Analysis 1.0 (MEGAX). Sequence logo was generated using WebLogo3 (http://weblogo.threeplusone.com/).

#### 2.3.3 Prediction of peptide-induced cytokine production

Peptide capability of inducing bystander cytokine production (IFN-γ and IL-4) from Th1 and Th2 axis of immune response was predicted by IFNepitope software (https://webs.iiitd.edu.in/raghava/ifnepitope/scan.php) and IL4Pred (https://webs.iiitd.edu.in/raghava/il4pred/predict.php). In both cases, SVM based-methods were applied. Data regarding physicochemical features such as hydrophobicity, hydrophilicity, sterichinderance, net hydrogen, side bulk, charge, hydropathicity, isoelectric point (pI) and molecular weight, of peptides with complete or partial overlapping with YFV-17DD, was also acquired with IL4Pred.

### 2.4 Statistical analyses

Statistical analyses were performed using the GraphPad Prism v.8.0 software (GraphPad Software, California, USA). Non-parametric data distribution was confirmed by the Shapiro-Wilk test. Wilcoxon test was applied for statistical comparisons involving only two groups. Multiple comparisons were performed using the Kruskal-Wallis method followed by Dunn’s post-test, in the case of unpaired data, and the Friedman method with Dunn’s post-test to compare paired data. Chi square test was applied for the comparison of unpaired data regarding number of reactive peptides. Spearman’s rank correlation test was applied for the correlation of *in vitro* reactivity and *in silico* prediction of cytokine induction. Differences were considered statistically significant at p <0.05. Principal component analysis (PCA) was performed using Minitab 19.

## 3 Results

**Figure 1:**
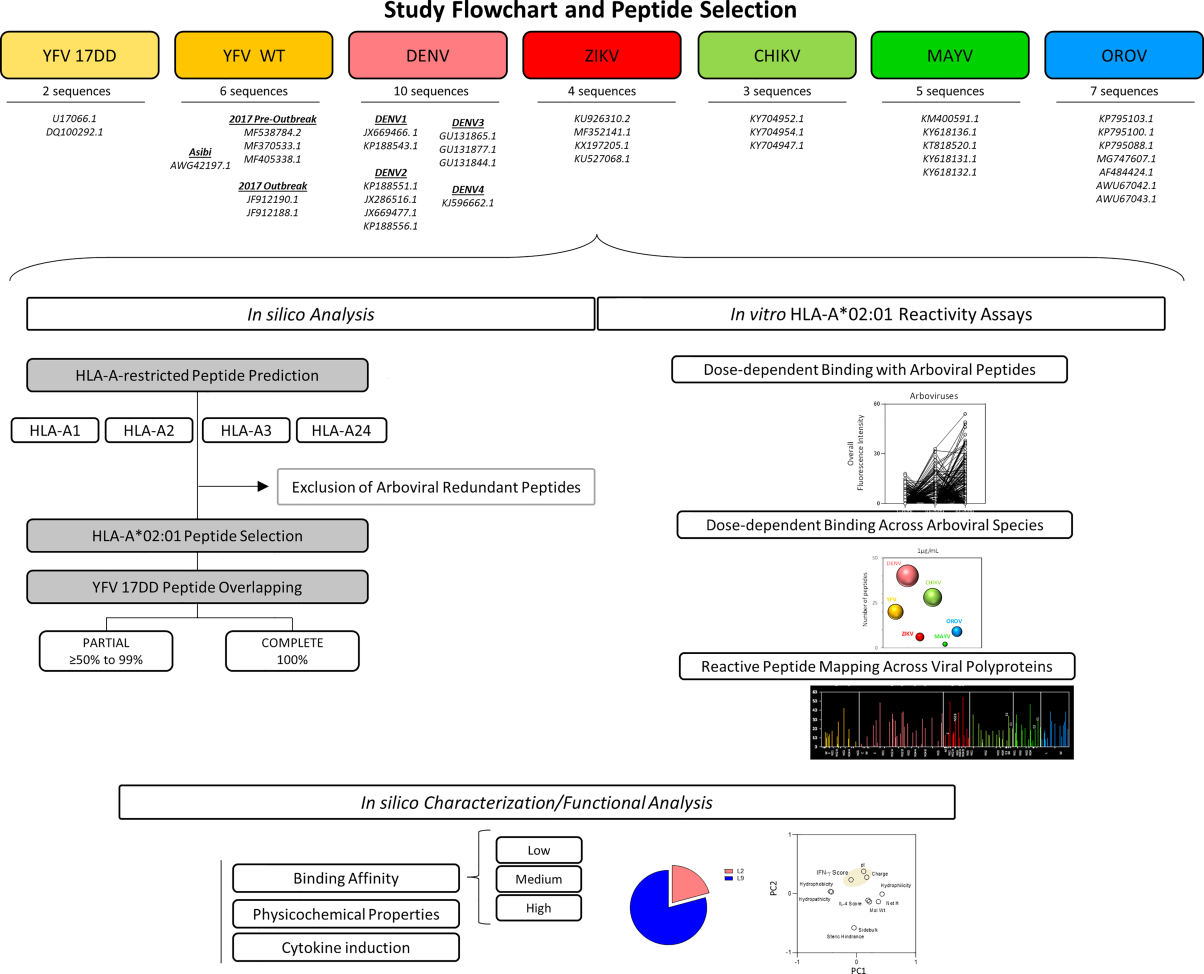
Flowchart illustrating Study design and Peptide Selection. Thirty-one sequences comprising 7 arboviruses and 10 serotypes were applied for assessing peptide signatures *in silico* for HLA-A1, A2, A3 and A24 MHC-I haplotypes. HLA-A*02 restricted-peptides from YFV wild strain, ZIKV, DENV 1-4, CHIKV, MAYV and OROV were selected and assessed for overlapping against YFV-17DD peptides. Biochemical parameters, prediction of IFN-γ and IL-4 induction, hydrophobicity, and the frequency of amino acid composition was measured for the identification of conserved residues.

To understand Arboviral epitope immunogenicity, a comprehensive *in silico* descriptive analysis of peptides from YFV, ZIKV, DENV 1-4, CHIKV, MAYV and OROV was performed. Detailed information on study design is displayed as a flowchart in **Figure 1**. Peptide prediction was performed considering four of the most prevalent major histocompatibility complex class I haplotypes in Brazil, namely, HLA-A1, HLA-A2, HLA-A3 and HLA-A24.

### 3.1 Peptide prediction for different MHC-I haplotypes indicates a clear prevalence of HLA-A*02:01-restricted peptides derived from arbovirus

**Figure 2:**
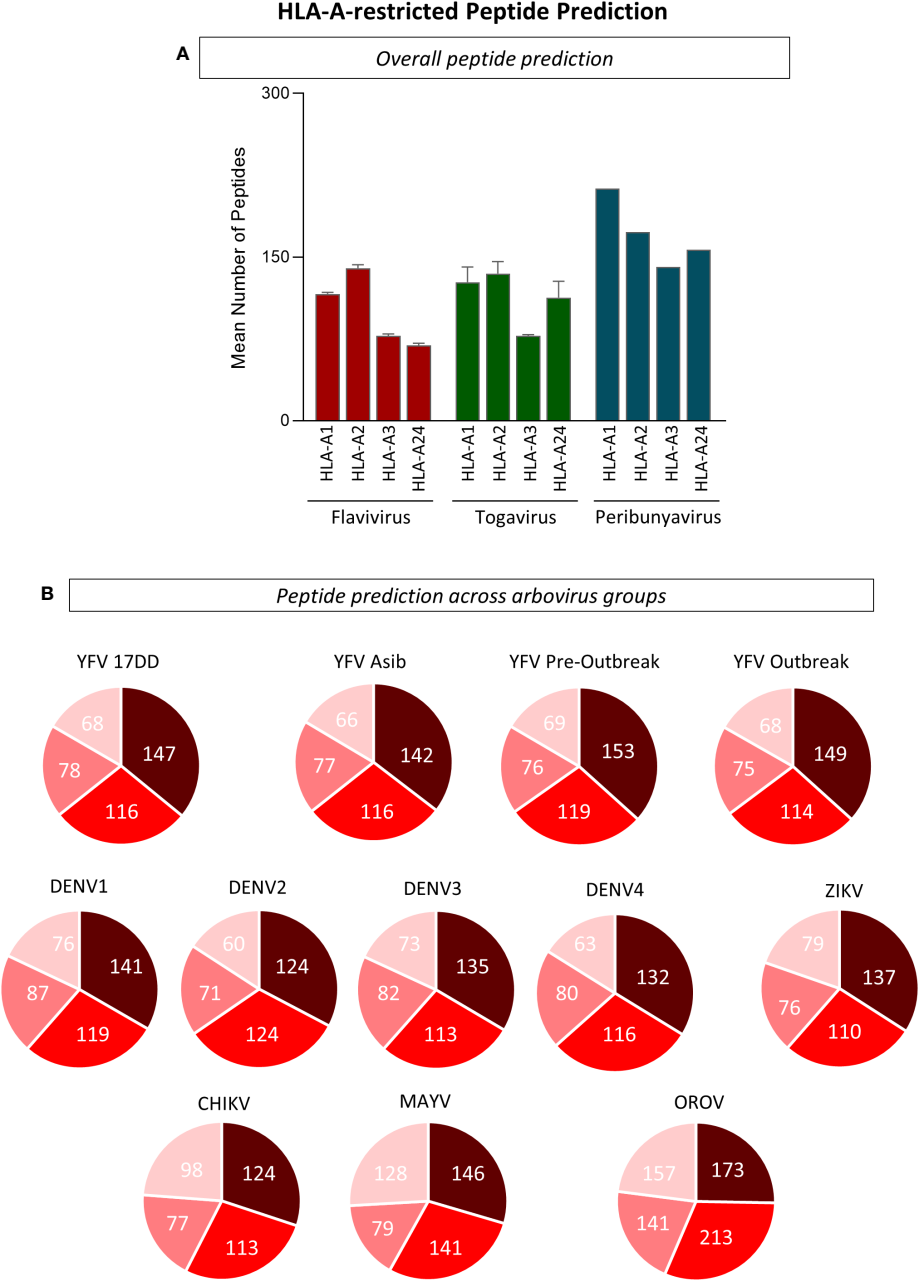
Evaluation of Peptide Restriction Regarding Human Leukocyte Antigen (HLA) Haplotype. **(A)** Number of restricted peptides predicted for HLA-A1, A2, A3 and A24 haplotypes using complete polyprotein sequences from Flavivirus, Togavirus and Peribunyavirus are expressed in bar graphs using mean plus standard deviation. **(B)** Pie charts for the number of HLA-restricted peptides for every arbovirus included in this study. Only peptides with combined prediction score ≥ 0.75 were considered in this analysis.

Immunodominance and haplotype restriction are very important parameters for the design of multiepitope proteins applicable to the differential diagnosis and vaccine development. The analysis which aimed at understanding *in silico* similarities and divergencies and HLA restriction for immunodominant arboviral peptides revealed that overlap amongst the arboviruses were found for peptide binders of different haplotypes: HLA-A1 (n=1,005), HLA-A2 (n=1,693), HLA-A3 (n=1,514), and HLA-A24 (n=999). A higher number of HLA-A*02:01-restricted peptides were predicted for YFV-17DD, YFV wild strains, DENV 1-4, ZIKV, CHIKV and MAYV, when compared to other HLA-A haplotypes. For OROV, peptide restriction to HLA-A3 was prevalent, however an elevated number of HLA-A*02-restricted peptides was also observed for this arbovirus (**Figure 2**). Considering this results, HLA-A*02-restricted peptides were selected for further evaluations.

### 3.2 Comprehensive *in silico* analysis of HLA-A*02:01-restricted peptide signature indicates poor overlapping between YFV-17DD and other arboviruses

**Figure 3:**
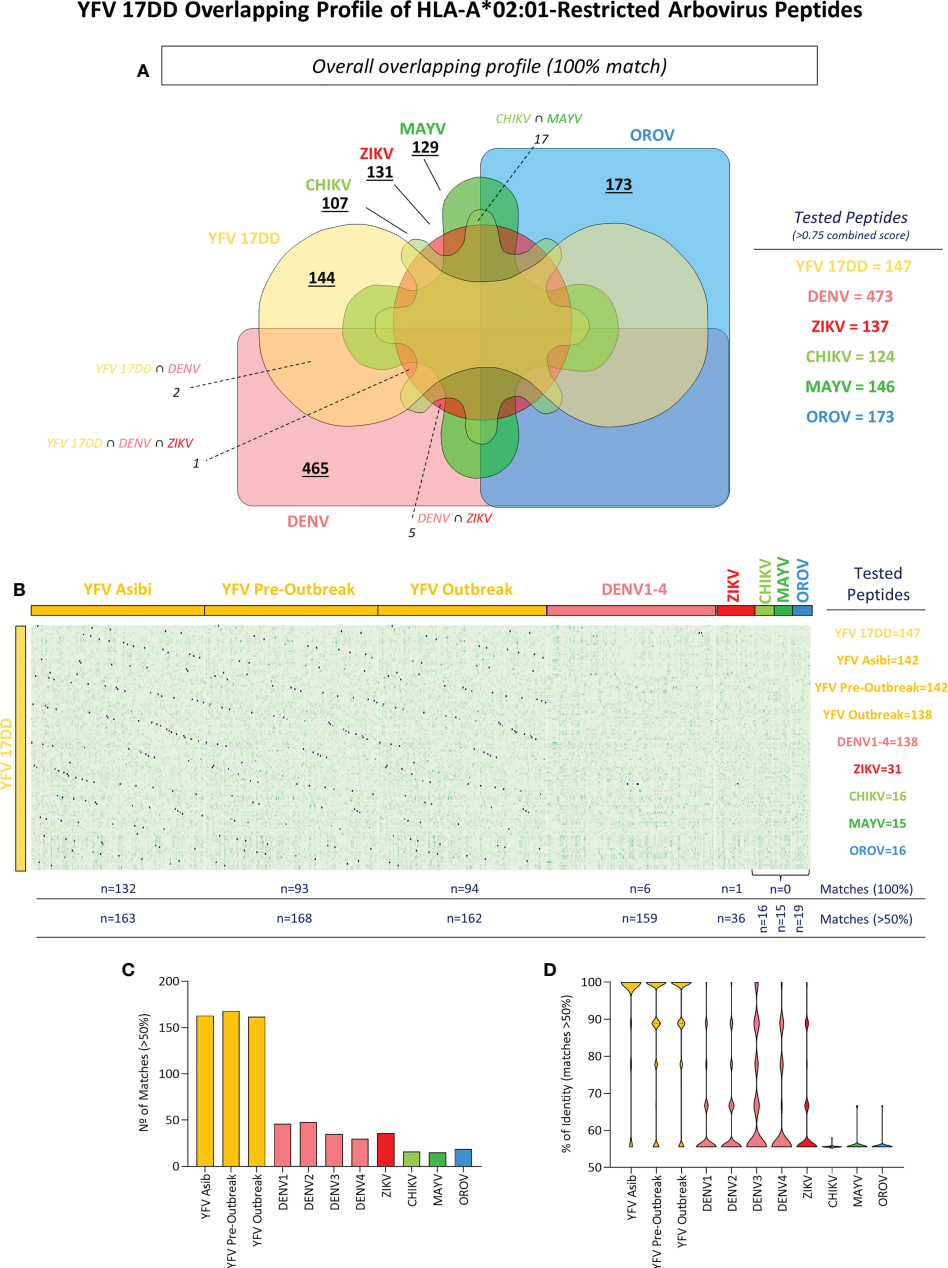
Identification of YFV-17DD Overlapping Peptides in Other Arboviruses and Assessment of HLA-A*02:01 Reactivity. **(A)** Venn diagram with shared peptides between all assessed arboviruses. Intersecting regions between arboviruses and YFV-17DD are indicated by dotted lines. **(B)** Heatmap analysis with percentage of identity between HLA-A*02:01-restricted peptides from arbovirus. Y axis indicated YFV-17DD peptides, X axis indicates peptides from other arboviruses. Only peptides with an identity rate ≥ 50% with peptides derived from YFV-17DD were considered **(C)** Number of matches with YFV-17DD peptides for all assessed arbovirus. **(D)** Violin plot with percentage of overlapping peptide’s identity from all assessed arboviruses with YFV-17DD peptides.

Arboviral epitopes which overlap amongst viruses were found for peptide binders of different viral families. As demonstrated by **Figure 3**, overlapping was demonstrated by the number of peptides from YFV wild strains, ZIKV, DENV 1-4, CHIKV, MAYV and OROV that correspond to YFV-17DD peptides (namely the number of matches). After selection of HLA-A*02:01-restricted-peptides, an identity matrix between 17DD-YFV and ZIKV, DENV 1-4, CHIKV, MAYV and OROV was created to identify overlapping peptides. From over 1700 epitopes, only three YFV-17DD peptides with combined score ≥0.75 were common to one or more arbovirus. Namely, peptide YFV_17DD_1995_127 presented complete overlapping with DENV2_PE1_116 and DENV3_BR5_112; peptide YFV_17DD_1995_42 presented complete overlapping with DENV3_BR5_25, and peptide YFV_17DD_1995_76 presented complete overlapping with DENV1_SJRP1_62, DENV3_BR5_55, DENV4_62 and ZIKV_RN1_56. No epitopes predicted for CHIKV, MAYV or OROV were shared by YFV-17DD vaccinal strain (**Figure 3A**).

To expand the assessment of the peptide signature shared among the selected arboviruses and YFV-17DD, peptides exhibiting at least 50% of similarity with epitopes predicted for YFV-17DD were selected (**Figure 3B**). From 244 peptides matches of complete or partial overlapping (>50% identity), 195 belonged to other flavivirus (ZIKV n=36; DENV1 n=46; DENV2 n=48; DENV3 n=35; DENV4 n=30), 31 were from togaviruses (CHIKV n=16; MAYV n=15) and 19 matched peptides were found for OROV (peribunyavirus). As expected, wild strains from yellow fever virus presented an elevated number of matched peptides with 17DD vaccinal strain (YFV Asib n=163; YFV Outbreak n=162; YFV Pre-Outbreak n=168) (**Figure 3C**). The same tendency was observed for identity average. Peptides from ZIKV and DENV serotypes 1-4 had between 61.15% (DENV1) and 67.65% (DENV3) of similarity with YFV-17DD peptides. Means of identity for CHIKV, MAYV and OROV remained close to baseline, ranging from 55.60% (CHIKV) to 56.18%. These observations were a clear contrast to peptides from YFV wild strains, that presented average identity close to 90% (93% in YFV Asib; 89.65% in YFV Outbreak and 89.36% in YFV Pre-Outbreak), which can be further observed as the noticeable inversion of the violin plot (**Figure 3D**). Other prediction parameters were also evaluated and are shown in **Supplementary Figure 1**.

### 3.3 Peptide microarray indicates dose-response effect of peptide reactivity to an HLA-A2:β2M:Ig protein

**Figure 4:**
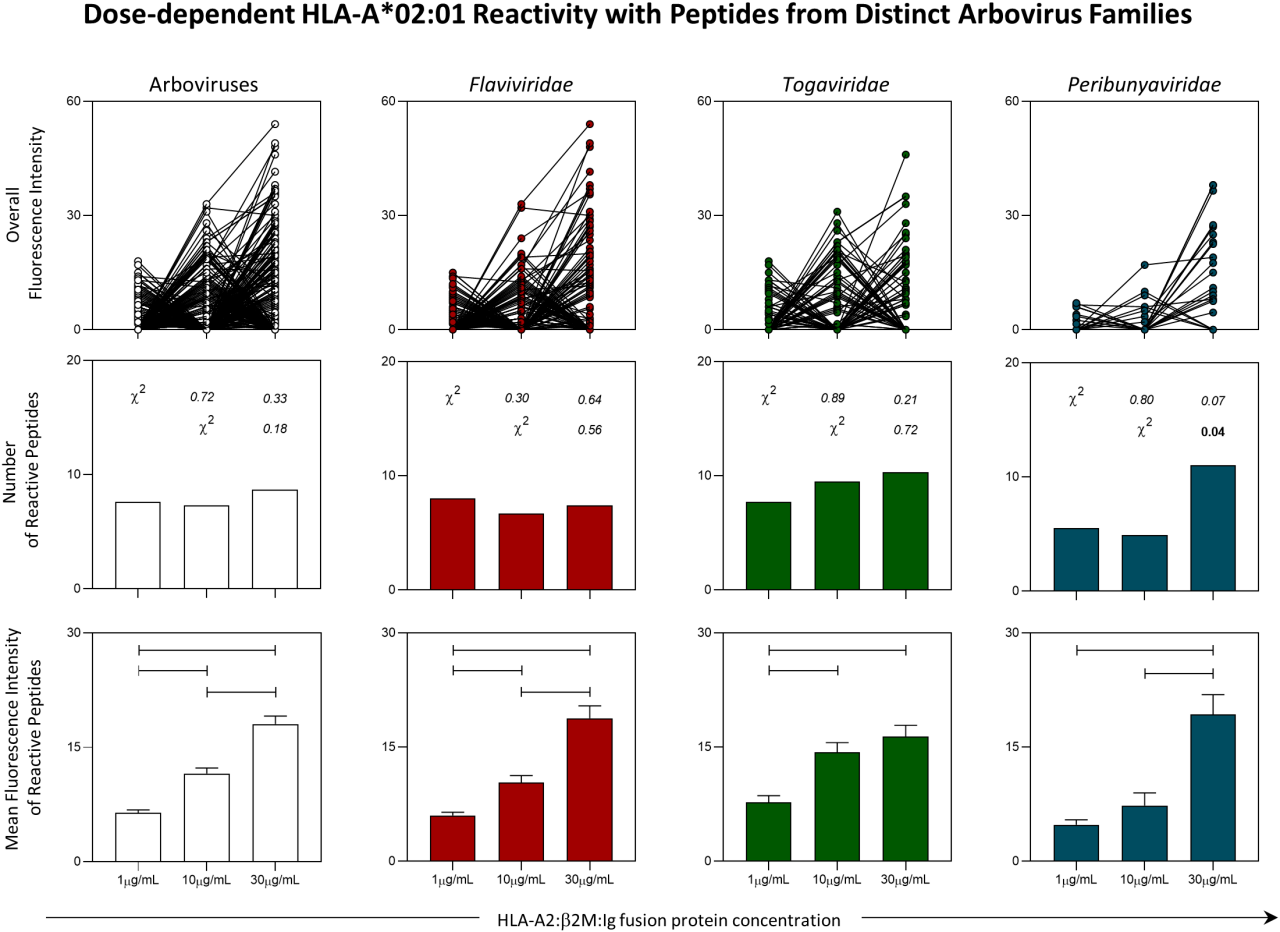
Panoramic Evaluation of HLA-A*02:01 Reactivity for Peptides Derived from Arbovirus. Peptide microarray was performed with an HLA-A*02:01 dimeric protein in three concentrations (1µg/mL, 10µg/mL and 30µg/mL). Overall fluorescence of peptide-HLA-A*02:01 reactivity (top panels), absolute number of HLA-*02:01-reactive peptides (middle panels) and mean of fluorescence intensity of HLA-*02:01-reactive peptides (lower panels) were evaluated. Chi-square test (χ2) was used to compare the number of HLA-A*02:01-reactive. Obtained p values are indicated above each bar. Comparisons regarding the mean of fluorescence intensity of HLA*02:01-reactive peptides were performed using the Kruskal-Wallis. Statistical significances were considered for p < 0.05 and are indicated by connecting lines.

Intending to extend on the understanding of immunodominant peptides from Arbovirus, *in vitro* assessment of peptide-HLA-A*02:01 reactivity was evaluated. For that, a peptide microarray was designed and generated as shown in **Supplementary Figure 2** (PEPperPRINT^©^, Heidelberg, Germany). The peptide array employed fixed concentrations of peptides (1nM) and three different concentrations (1μg/mL, 10μg/mL or 30μg/mL) of a dimeric protein containing HLA-A*02:01 domain fused to β2-microglobulin (HLA-A2:β2M:Ig Protein) produced in house. Global analysis of the fluorescence intensity for peptide-HLA-A*02:01 binding indicated a dose-response effect regarding the concentration of the HLA-A*02:01 dimeric protein with fixed concentrations of peptide in the array. The same pattern was observed for peptides from all arboviruses taken together and also for peptides classified by viral families (*Flaviviridae*: YFV, DENV1-4 and ZIKV; *Togaviridae*: CHIKV and MAYV; *Peribunyaviridae*: OROV), as indicated in the upper panels of **Figure 4**.

The absolute number of HLA-A*02:01-reactive peptides in all three concentrations of HLA-A*02:01 dimeric protein remained stable in assessment of all arboviruses taken together and for viruses belonging to the *Flaviviridae* and *Togaviridae* families. For OROV, it was possible to observe an increase in the number of reactive peptides in the concentration of 30μg/mL as compared to a concentration of 10μg/mL of HLA-A*02:01 dimeric protein (**Figure 4** - middle panels).

Despite the stability in the number of HLA-A*02:01-reactive peptides in two of the three viral families analyzed, the trend of the dose-response effect was confirmed by the evaluation of the mean fluorescence intensity. In this case, there was a progressive increase in the reactivity of peptides derived from arboviruses analyzed together and for peptides derived specifically from the *Flaviviridae* family. As for the peptides from *Togaviridae* family, the reactivity was higher for the concentrations of 10μg/mL and 30μg/mL, but no difference was observed between these two concentrations. As for peptides derived from OROV (*Peribunyaviridae:family*), the highest reactivity was observed for the concentration of 30μg/mL of HLA-A*02:01 dimeric protein (**Figure 4** - lower panel).

### 3.4 Non-overlapping immunodominant epitopes display the highest HLA-A*02:01 affinity binding assessed *in vitro*

**Figure 5:**
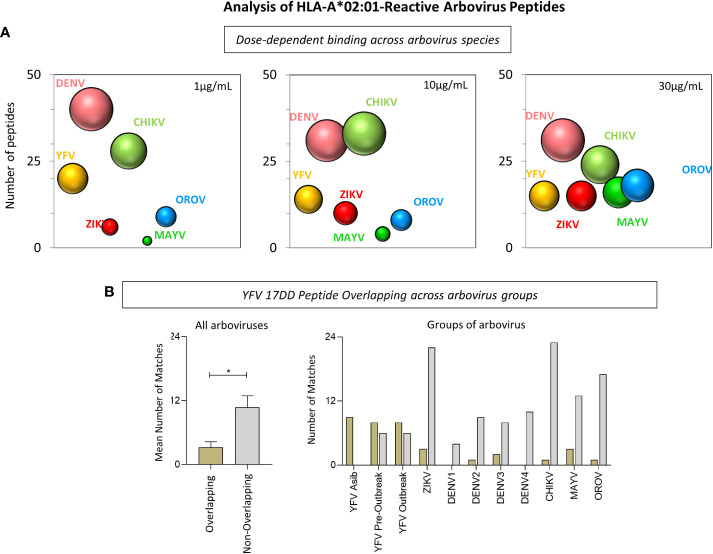
Analysis of HLA-A*02:01-Reactive Arbovirus Peptides. **(A)** Bubble size represents the number of HLA-A*02:01-reactive peptides at 1µg/mL, 10µg/mL and 30µg/mL of HLA-A*02:01 dimeric protein for each arbovirus included in this study. **(B)** Number of HLA-A*02:01-reactive peptides with and without overlapping YFV-17DD for all arboviruses (left) and for groups of arboviruses (right).

Quantitative analysis of HLA-A*02:01-reactive peptides derived from arboviruses were further evaluated and demonstrated that non-overlapping immunodominant epitopes display the highest HLA-A*02:01 affinity binding assessed *in vitro*. First, data analysis demonstrated that DENV strains show the highest number of reactive peptides among Flavivirus for all three HLA-A*02:01 dimer concentrations (**Figure 5A**). Considering all arbovirus sequences studied, the number of DENV-derived peptides with HLA-A*02:01 reactivity was the highest in two of the three HLA-A*02:01 dimer protein concentrations, mainly at 30μg/mL. In addition, CHIKV, next to DENV, presented the highest number of reactive peptides at 10μg/mL (**Figure 5A**). On a second note, **Figure 5B** shows that a significant higher number of non-overlapping peptides presented reactivity when compared to YFV-17DD overlapping peptides. This was observed for peptides derived from all assessed arboviruses except for YFV wild strain derived peptides, for which most of HLA-A*02:01 reactive peptides presented species-specific overlapping with YFV-17DD sequences, as expected (**Figure 5B**).

**Figure 6:**
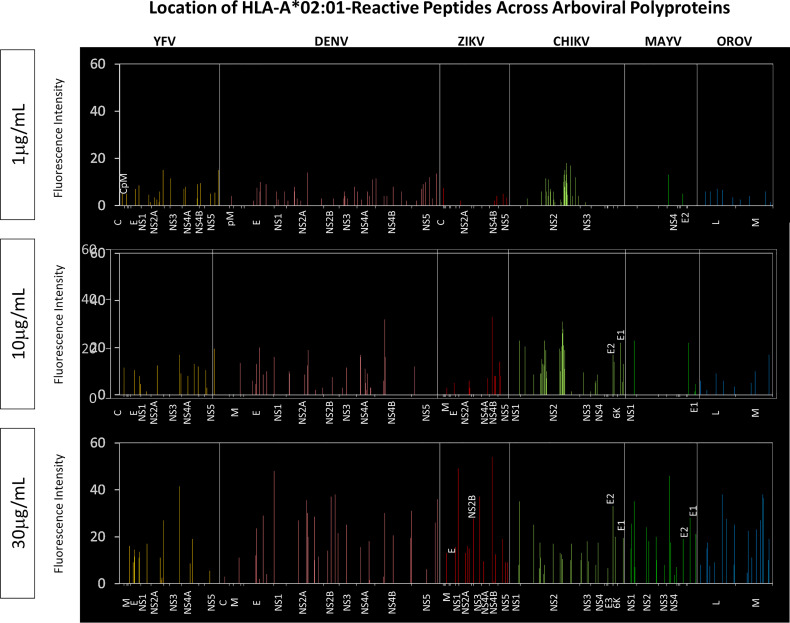
Location of HLA-A*02:01-Reactive Peptides Across Arboviral Polyproteins. Multiple alignment using MUSCLE algorithm was performed using HLA-A*02:01-reactive peptides and the respective arbovirus polyprotein. Reactive peptides from all three HLA-A*02:01 protein concentrations were assessed separately. Only regions with HLA-A*02:01-reactive peptides are identified on the x-axis of the graph for every arbovirus evaluated.

Of note, the assessment of HLA-A*02:01-reactive peptides across virus polyproteins highlighted non-structural proteins as “hot-spots” for HLA-A*02:01-restricted peptides (**Figure 6**). The identification of HLA-A*02:01-reactive peptides along viral polyproteins indicated that non-structural proteins encompass the highest HLA-A*02:01-restricted peptides binders. This effect was observed when peptides reacted at all three concentrations of HLA-A*02:01 dimeric protein. The global fluorescence intensity for HLA-A*02:01 binding indicated a significant increase in the reactivity of peptides for non-structural protein in a dose-dependent manner as indicated in **Figure 6**.

### 3.5 Non-structural proteins 2 and 4 are important hydrophobic sites in YFV-17DD

**Figure 7:**
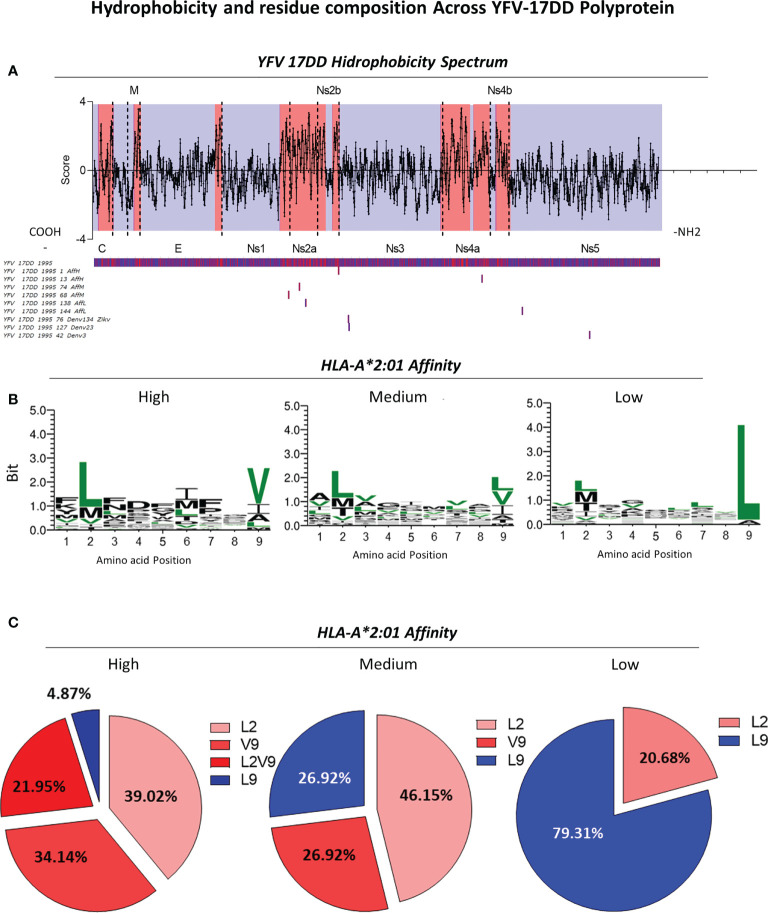
Assessment of Hydrophobicity Across YFV-17DD Polyprotein and Conserved Residues in Overlapping Peptides with High, Medium and Low HLA-A*02:01 predicted affinity. **(A)** Hydrophobicity values for YFV-17DD complete polyprotein. Regions of high hydrophobicity are indicated in red and of low hydrophobicity are indicated in blue. The three peptides with complete YFV-17DD overlapping as well as peptides of high, medium and low HLA-A*02:01 affinity score were aligned with the YFV-177 polyprotein using MUSCLE algorithm. **(B)** Logo sequence analysis using two representative peptides of each arbovirus in each HLA-A2 affinity range (high, medium and low HLA-A2 affinity score). Conserved motifs are indicated in (green). **(C)** Frequency of leucine and valine in positions 2 and/or 9 for all three HLA-A2 affinity ranges.

Hydrophobicity of peptides is a crucial parameter to understand protein binding affinity (30–32). Therefore, assessment of hydrophobicity was performed for the YFV-17DD polyprotein in order to understand the influence/correlation of this parameter with immunodominance results observed *in silico* and *in vitro*. Data analysis indicated the presence of major hydrophobic sites in the final segment of non-structural protein 1 throughout 2a (Ns2a) and in non-structural proteins 2b (Ns2b), 4a (Ns4a) and 4b (Ns4b). Conversely, structural proteins from YFV-17DD such as the capsid and membrane presented only restricted areas with elevated hydrophobicity (**Figure 7A**). The establishment of global median for HLA-A*02:01-peptide binding affinity using *in silico* score allowed the identification of peptides with high, medium and low affinity score values in all arboviruses. The alignment of representative YFV-17DD peptides with different ranges of HLA-A*02:01 predicted *in silico* binding scores revealed that peptides with high and medium affinity scores are derived from non-structural Ns2b (YFV-17DD_1995_1), Ns4a (YFV-17DD_13 and YFV-17DD_1995_74), and Ns1 (YFV-17DD_1995_68). As for the peptides with low HLA-A*02:01 affinity scores, these sequences were derived from YFV Ns3a and Ns5 (**Figure 7A**). Regarding peptides with complete overlap amongst YFV-17DD and other flaviviruses, two peptides were derived from Ns3 (YFV-17DD_1995_7, observed in DENV1, DENV3, DENV4 and ZIKV; and YFV-17DD_1995_127, common to DENV2 and DENV3) and one peptide was derived from the Ns5 portion (YFV-17DD_1995_42, also found in DENV3) (**Figure 7A**).

For the assessment of amino acid patterns within peptides of different HLA-A*02:01 affinity scores, two sequences of high, medium and low affinity scores were aligned for the identification of a consensus sequence, highlighting all conserved residues among the assessed arboviruses (**Figure 7B**). For peptides with high HLA-A*02:01 affinity score, the presence of leucine in position two and valine in position nine were predominant. As for sequences with relative low HLA-A*02:01 affinity score, a remarkable presence of leucine in position 9 was observed (**Figures 7B, C**), occurring in 23 of the 24 aligned peptides (**Supplementary Figure 3**). A distinct pattern of conserved amino acids was not observed for peptides with medium HLA-A*02:01 predicted affinity score. However, sequence logo analysis allowed the identification of an intermediary profile, with a slight increase of leucine in the ninth position and a decreased proportion of this same amino acid in the second amino acid position (**Figure 7B**). Only peptides with high score of HLA-A2 binding presented both leucine in the second position and valine in the ninth position simultaneously, in a frequency of 37.50% (**Figure 7C**).

### 3.6 Predicted Th1 and Th2 immune response induced by arboviral peptides

**Figure 8:**
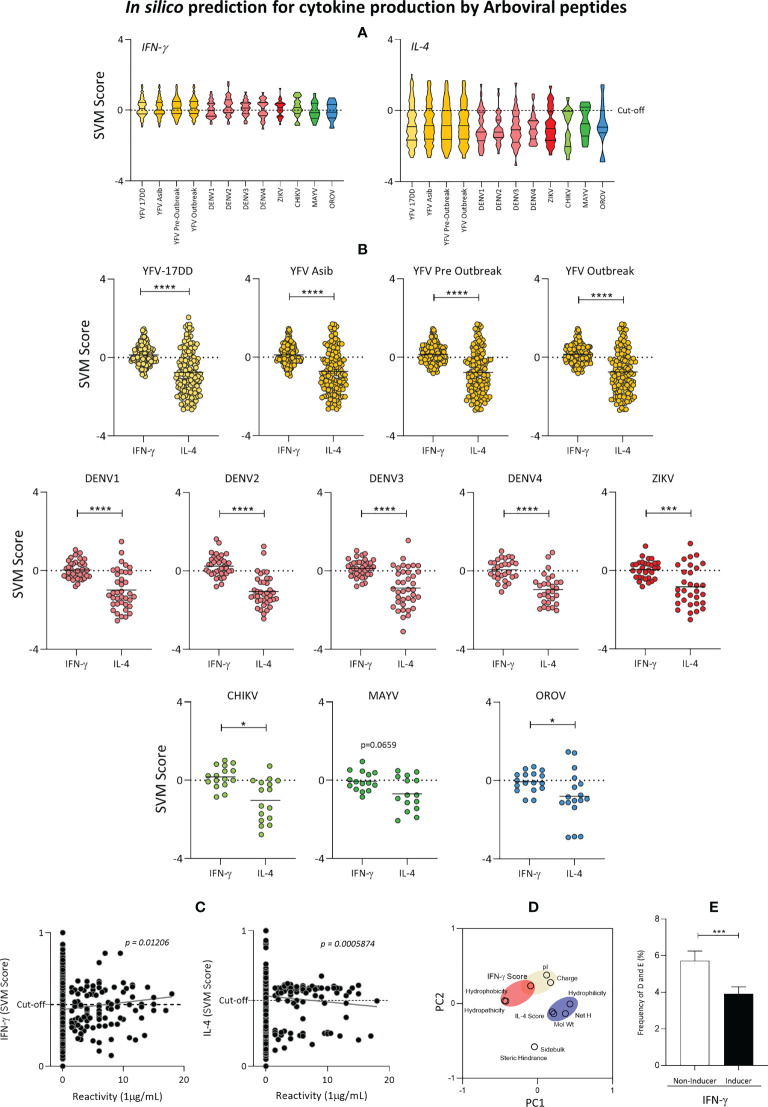
Cytokine induction by YFV-17DD Overlapping Peptides. **(A)** IFN-γ and IL-4 production score was assessed by *in silico* analysis for the peptides from arbovirus. **(B)** Comparison of IL-4 and IFN-γ induction scores within each assessed arbovirus. Dots indicate individual peptides red lines indicate mean with standard deviation. Statistical significances are indicated by asterisks (* = p<0.01; *** = p<0.0001; **** = p<0.00001). **(C)** Correlation between cytokine production scores and peptide-HLA-A*02:01 reactivity obtained *in vitro* was performed for IFN-γ (left panel) and IL-4 (right panel) by Spearman’s rank test. **(D)** Principal Component Analysis (PCA) with physicochemical properties of overlapping peptides and *in silico* scores for cytokine induction. Parameters related to IFN-γ induction are clustered in red and in blue for IL-4. **(E)** Frequency of acid residues [aspartic **(E)** and glutamic **(D)** acid] in IFN-γ inducer and non-inducer peptides identified by *in silico* evaluation.

Next, the predicted ability to induce cytokine production induced by peptides with complete or partial overlap with YFV-17DD, as well as peptides from the YFV vaccinal strain was also evaluated. For that, Interferon-gamma (IFN-γ) and Interleukine-4 (IL-4) predicted scores were calculated and contrasted amongst viral families. Data analysis showed that induction of IFN-γ was notably higher among peptides from almost all assessed arboviruses. Only DENV1, MAYV and OROV presented negative median values (cut-off = 0) (**Figure 8A**). IL-4 induction by peptides of all arboviruses presented negative median values. Additionally, ZIKV, DENV 1-4, CHIK and OROV presented 75% of peptides with negative valued score for IL-4 induction (**Figure 8A**).

Comparative analysis of IL-4 and IFN-γ induction within viruses confirmed that peptides from all arboviruses presented a substantial bias of peptides towards Th1 IFN-γ immune response axis. Overlapping peptides followed the same trend. This observation is confirmed by the fact that all three peptides common to YFV vaccinal strain and one peptide from Flavivirus family were predicted as IFN-γ inducers but not IL-4 inducers (**Figure 8B**). Overall, peptide-HLA-A*02:01 *in vitro* reactivity was directly correlated to IFN-γ score (p=0.01206; R=0.005365) and inversely correlated to IL-4 score (p=0.0005874; R=0.004536), as displayed in **Figure 8C**. In addition, principal component analysis (PCA) showed that cytokine induction may be triggered according to peptide biochemical properties. While peptide charge, isoelectric points and hydrophobicity seem to cluster closer to the peptide IFN-γ induction power, IL-4 induction seems to be closely related to peptide hydrophilicity, molecular weight and net hydrogen (**Figure 8D**). Considering that peptide charge is influenced by acid residues, frequencies of aspartic acid (D) and glutamic acid (E) was assessed in peptides capable of inducing IFN-γ production. The results confirmed that peptides considered to be IFN-γ inducers presented lower frequencies of aspartic acid (D) and glutamic acid (E) when compared with non-inducer peptides (**Figure 8E**).

## 4 Discussion

This study presents a comprehensive overview of immunodominant peptides for specific CD8^+^ T-cell responses targeting peptides of circulating wild type and 17DD Yellow Fever virus as well as ZIKV, DENV (serotypes 1-4), CHIKV, MAYV, and OROV. Immunodominant HLA-A2-restricted peptides from Yellow Fever virus Asib strain were used as control group giving its proximity with the vaccinal strain (33, 34). Wild strains from before and after the 2016-2019 Brazilian Yellow Fever outbreak were also included in all analysis to provide reliable control groups (35).

Interaction between peptide/MHC-I complex and TCR is the first crucial step to CD8^+^T cell activation. However different MHC-I haplotypes are prone to present distinct assortments of peptides with different anchor residues based on binding cleft sequence. Such variety affects quaternary structure of peptide/MHC-I complex, which leads to different TCR recognition and can affect cellular immune response (36–41). Among the four haplotypes tested, a higher number of HLA-A*02-restricted peptides were observed for all tested arbovirus except for OROV. In addition to the high prevalence in Brazilian population and the superior number of restricted peptides, previous studies have showed that peptide binding affinity with HLA-A*02 haplotypes can be correlated with immunogenicity, which also contributed to the selection of HLA-A*02 restricted peptides to further analysis (42–44).

There was no presence of concomitant HLA-A*02-restricted peptides between 17DD and CHIKV, MAYV or OROV. A result somewhat expected, considering the phylogenetic distance of Flavivirus (YFV), Togavirus (CHIKV and MAYV) and Peribunyavirus (OROV) (45). However, a poor overlap of HLA-A*02-restricted immunodominant peptides was also observed among 17DD and other arbovirus from the Flaviviridae family, such as ZIKV and DENV (serotypes 1-4). The GVFHTMWHV 17DD peptide was observed for ZIKV, DENV1, DENV3 and DENV4; TMWHVTRGA was common to 17DD, DENV2 and DENV 3; and YMWLGARYL, to 17DD and DENV3. This low number of matching peptides does not seem to follow the percentage of identity between complete polyprotein amino acid sequence of these virus, once all DENV serotypes present identity levels over 45% with 17DD and ZIKV reaches 46% of 17DD identity (data not shown).

Peptides with partial overlap were observed for all 5 arbovirus and 8 serotypes here analyzed. The number of partial overlapping peptides and rates of shared amino acids followed phylogenetic distance, but the means of peptide identity with 17DD remained particularly low for ZIKV and serotypes 1, 2, 3 and 4 of DENV, with numbers closer to those observed for CHIKV, MAYV and OROV than for YFV wild strains and YFV Asib strain. While all three peptides with complete overlapping were considered to be immunogenic, but with relative low scores, more than 40% of the partial overlapping peptides from ZIKV, DENV (serotypes 1-4), CHIKV, MAYV and OROV were not considered to be reactive at all. These results highlight how truly deficient the overlapping peptides may be in inducing broad antiviral CD8^+^ T responses effective concomitantly to 17DD and other arboviruses (40, 41).

In regard to the amino acid patterns, in agreement with our findings, peptide positions 2 and 9 seen to be important anchoring sites for HLA-A*02 binding, with leucine (L) and valine (V) playing important roles in peptide affinity when observed in those respective positions. Not only that, but the occurrence of leucine (L) as last residue on sequences with low affinity score also corroborates with previous found data (38). The overall prevalence of non-polar amino acids in the aligned peptides agrees with several findings associating peptide hydrophobic residues not only with MHC-I binding but also with induction of CD8^+^ T response (30, 32, 46).

In this regard, the next step was to perform a global assessment of hydrophobicity throughout the entire length of 17DD polyprotein sequence. Areas of predominant hydrophobicity were found in non-structural protein 2a (Ns2a) and 2b (Ns2b) as well as non-structural protein 4a (Ns4a) and 4b (Ns4b). The alignment of the polyprotein sequence with 17DD peptides of different HLA-A*02 affinity, showed that peptides with high and medium HLA-A*02 affinity binding are precisely from these more hydrophobic sites of the polyprotein. In addition, all three 17DD peptides with complete overlap with ZIKV and/or DENV serotypes are from less hydrophobic sites, such as Ns3 and Ns5 protein.

To date, there is little information about the immunogenicity of specific portions of 17DD polyprotein. Nonetheless, non-structural protein NS4b have been described as highly immunogenic for vaccinal strains of Yellow Fever vaccine in several studies, with the identification of several HLA-A*02-restricted peptides and persistence of long-lasting effector and central memory CD8^+^ T cell responses targeting Ns4b peptides (44, 47–49). Data regarding other non-structural proteins such as Ns1 and Ns2a and Ns2b are still scarce, but few immunogenic peptides have also been described by Lund and colleagues, agreeing with our findings (44). The already established importance of hydrophobic residues for the interaction between peptides and TCR is yet another support for the importance of these non-structural protein in the activation and maintenance of cellular antiviral response (32).

The 17DD vaccination triggers an early and late activation of both CD4^+^ and CD8^+^ T cell responses, with Th1 and Th2 axis of immune response highly associated to protection (5, 50–54)). Considering the rich peptide microenvironment during antigen presentation and the importance of cytokines in this process, an overall assessment of the capability of HLA-A*02-restricted immunodominant peptides to induce production of IFN-γ and IL-4 through peptide-MHC-II binding was also assessed by bioinformatic tools (55). Interestingly, there was a predominance of IFN-γ inducers in comparison with IL-4 for all arboviruses. Little to no difference was observed in the induction of these cytokines among 17DD peptides and the other arboviruses. This is to be expected given the important antiviral role of IFN-γ and the pre-selection of immunodominant peptides with combined score above 0.75 (56, 57). IFN-γ is a major proinflammatory cytokine produced by T cells which is important for cytotoxic antiviral responses as well in the maturation of antigen presenting cells (58–61). Integrative assessment of peptide biochemical features such as hydrophobicity and hydrophilicity and the induction of immune response from the Th1 or Th2 axis represented by induction of IFN-γ or IL-4 production was carried out using principal component analysis (PCA). In this case, we observer a close relation of peptide hydrophilicity with IL-4 induction. Despite scarce information regarding this association with viral peptides, our findings are in accordance with Quintilio et al., and their observations regarding induction of Th2 response in the form of IgG1 production by hydrophilic vaccinal adjuvants for H1N1 such as monophosphoryl-lipid A (MPLA), alum and riboflavin (B2 vitamin) (62).

Closer relation of Th1 axis with peptide isoelectric point is probably related to the lowest occurrence of acid residues peptides considered inductors of IFN-γ production. There are no reports of this association for peptides derived from arbovirus, but a decrease in CD8^+^ T cell activation and cytotoxicity was observed for tumoral peptides after substitution of serine (S), an uncharged amino acid, for aspartic (D) or glutamic acid E (63).

Taken together, these comprehensive results generated by *in silico* and high-throughput microarray peptide analysis show not only a poor overlapping of HLA-A*02-restricted immunodominant peptides among 17DD and arboviruses such as ZIKV, DENV (serotypes 1-4), CHIKV, MAYV and OROV but also suggests that the shared peptides between YFV-17DD and the mentioned arboviruses are possibly uncapable of inciting effective and lasting CD8^+^ T cell response. More *in vitro* and *in vivo* studies are still needed to confirm these findings. This study also creates a panoramic overview regarding MHC-class I-restricted immunodominant peptide mapping from several arboviruses of medical importance in Brazil and could shed light onto the design of putative novel vaccines for virus-specific arboviral infections.

## Data availability statement

The datasets presented in this study can be found in online repositories. The names of the repository/repositories and accession number(s) can be found in the article/**Supplementary Material**.

## Author contributions

Designing research study: JC-d-R; supervised research project and acquired funding: VP-M and JC-d-R; conducting experiments: ÁLR, FA, PO, LT, and GF; acquiring data: ÉL, ÁL, AL, and AV; analyzing data: ÁL, ÉL, OM-F, VP-M, and JC-d-R; advisory committee: FF, EB-S, and MT; provided reagents and funding: FF, EB-S, MT, VP-M, and JC-d-R; writing the manuscript: ÁL, OM-F, VP-M, JC-d-R. VP-M, and JC-d-R are corresponding authors. All authors contributed to the article and approved the submitted version.

## Funding

This work was supported by Fundação de Amparo à Pesquisa de Minas Gerais (FAPEMIG, APQ-01499-21), Conselho Nacional de Desenvolvimento Científico e Tecnológico (CNPq MCTI/CNPQ/Universal 14/2014 – A Tier; process# 458134/2014-7) and Coordenação de Aperfeiçoamento de Pessoal de Nível Superior (CAPES). FF, EB-S, OM-F, and JC-d-R received PQ fellowships from CNPq.

## Acknowledgments

We thank colleagues from the Basic and Applied Virology Lab and Biomarkers Research Group for insightful comments. We thank Dr. Silvia Giannattasio Ferraz for technical support and bioinformatics assistance. We also thank the Monoclonal Antibody development Flow Cytometry facility and staff for initial technical support. We thank Dr. Gabriela de Melo Franco and Dr. Danielle Soares de Oliveira Daian e Silva for additional lab assistance.

## Conflict of interest

The authors declare that the research was conducted in the absence of any commercial or financial relationships that could be construed as a potential conflict of interest.

## Publisher’s note

All claims expressed in this article are solely those of the authors and do not necessarily represent those of their affiliated organizations, or those of the publisher, the editors and the reviewers. Any product that may be evaluated in this article, or claim that may be made by its manufacturer, is not guaranteed or endorsed by the publisher.

## References

[1] Viana L Pimenta C Araújo E Teófilo T Costa T Costa K. Reemerging arboviruses: clinical-epidemiological profile of hospitalized elderly patients. arboviroses reemergentes: perfil clínico-epidemiológico de idosos hospitalizados. Rev da Escola Enfermagem da U S P (2018) 52:1–7:e03403. doi: 10.1590/S1980-220X2017052103403 30517293

[2] Vieira DS Zambenedetti MR Requião L Borghetti IA Luna L Rampazzo R. Epidemiological profile of zika, dengue and chikungunya virus infections identified by medical and molecular evaluations in rondonia, Brazil. Rev do Instituto Med Trop Sao Paulo (2019) 61:1–6:e40. doi: 10.1590/S1678-9946201961040 PMC6710006 31432989

[3] World Health Organization. Data from: Weekly epidemiological record: Dengue vaccine: WHO position paper – September 2018 (2018). Available at: https://www.who.int/publications/i/item/who-wer9335-457-476 (Accessed October 2022).

[4] da Costa-Rocha IA Campi-Azevedo AC Peruhype-Magalhães V Coelho-Dos-Reis JG Fradico JRB Martins-Filho AO. Duration of humoral and cellular immunity 8 years after administration of reduced doses of the 17DD-yellow fever vaccine. Front Immunol (2019) 10:1211. doi: 10.3389/fimmu.2019.01211 31293563 PMC6598206

[5] Costa-Pereira C Campi-Azevedo AC Coelho-Dos-Reis JG Peruhype-Magalhães V Araújo MSS Martins-Filho AO. Multi-parameter approach to evaluate the timing of memory status after 17DD-YF primary vaccination. PloS Negl Trop Dis (2018) 12(6):1–17:e0006462. doi: 10.1371/journal.pntd.0006462 PMC5991646 29879134

[6] Campi-Azevedo AC Reis LR Peruhype-Magalhães V Coelho-Dos-Reis JG Antonelli LR Martins-Filho AO. Short-lived immunity after 17DD yellow fever single dose indicates that booster vaccination may be required to guarantee protective immunity in children. Front Immunol (2019) 10:2192. doi: 10.3389/fimmu.2019.02192 31616412 PMC6775283

[7] Campi-Azevedo AC Peruhype-Magalhāes V Coelho-Dos-Reis JG Antonelli LR Costa-Pereira C Martins-Filho OA. 17DD yellow fever revaccination and heightened long-term immunity in populations of disease-endemic areas, Brazil. Emerg Infect Dis (2019) 8:1511–21. doi: 10.3201/eid2508.181432 PMC6649311 31298654

[8] Ahmed R Akondy RS. Insights into human CD8(+) T-cell memory using the yellow fever and smallpox vaccines. Immunol Cell Biol (2011) 89(3):340–5. doi: 10.1038/icb.2010.155 21301482

[9] Collaborative group for studies on yellow fever vaccines. Duration of post vaccination immunity against yellow fever in adults. Vaccine (2014) 39:4977–84. doi: 10.1016/j.vaccine.2014.07.021 25090646

[10] Ferdous S Kelm S Baker TS Shi J Martin ACR. B-cell epitopes: Discontinuity and conformational analysis. Mol Immunol (2019) . 114:643–50. doi: 10.1016/j.molimm.2019.09.014 31546099

[11] Felix AC Souza NCS Figueiredo WM Costa AA Inenami M Romano CM. Cross reactivity of commercial anti-dengue immunoassays in patients with acute zika virus infection. J Med Virol (2017) 89(8):1477–9. doi: 10.1002/jmv.24789 28229481

[12] Kikuti M Tauro LB Moreira PSS Campos GS Paploski IAD Ribeiro GS. Diagnostic performance of commercial IgM and IgG enzyme-linked immunoassays (ELISAs) for diagnosis of zika virus infection. Virol J (2018) 15(1):108. doi: 10.1186/s12985-018-1015-6 30005683 PMC6045861

[13] Hurtado-Monzón AM Cordero-Rivera CD Farfan-Morales CN Osuna-Ramos JF De Jesús-González LA Ángel RM. The role of anti-flavivirus humoral immune response in protection and pathogenesis. Rev Med Virol (2020) 30(4):e2100. doi: 10.1002/rmv.2100 32101633

[14] Goncalvez AP Engle RE St Claire M Purcell RH Lai CJ. Monoclonal antibody-mediated enhancement of dengue virus infection *in vitro* and *in vivo* and strategies for prevention. Proc Natl Acad Sci USA (2007) 104(22):9422–7. doi: 10.1073/pnas.0703498104 PMC1868655 17517625

[15] Sanchez-Trincado JL Gomez-Perosanz M Reche PA. Fundamentals and methods for T- and b-cell epitope prediction. J Immunol Res (2017) 2017:1–15:2680160. doi: 10.1155/2017/2680160 PMC5763123 29445754

[16] Rammensee HG Falk K Rötzschke O. Peptides naturally presented by MHC class I molecules. Annu Rev Immunol (1993) 11:213–44. doi: 10.1146/annurev.iy.11.040193.001241 8476560

[17] Singh H Ansari HR Raghava GP. Improved method for linear b-cell epitope prediction using antigen’s primary sequence. PloS One (2013) 8(5):1–8:e62216. doi: 10.1371/journal.pone.0062216 PMC3646881 23667458

[18] Van Regenmortel MH. What is a b-cell epitope? Methods Mol Biol (2009) 524:3–20. doi: 10.1007/978-1-59745-450-6_1 19377933

[19] Mishra N Caciula A Price A Thakkar R Ng J Lipkin WI. Diagnosis of zika virus infection by peptide array and enzyme-linked immunosorbent assay. mBio (2018) 9(2):1–16:e00095-18. doi: 10.1128/mBio.00095-18 PMC5844993 29511073

[20] Larsen MV Lundegaard C Lamberth K Buus S Lund O Nielsen M. Large-Scale validation of methods for cytotoxic T-lymphocyte epitope prediction. BMC Bioinf (2007) 8:424. doi: 10.1186/1471-2105-8-424 PMC2194739 17973982

[21] Li Pira G Ivaldi F Moretti P Manca F. High throughput T epitope mapping and vaccine development. J Biomed Biotechnol (2010) 2010:325720. doi: 10.1155/2010/325720 20617148 PMC2896667

[22] Fabreti-Oliveira RA Nascimento E Fonseca CG Santos MA. The heterogeneous HLA genetic composition of the Brazilian population and its relevance to the optimization of hematopoietic stem cell donor recruitment. Tissue Antigens (2014) 2:187–97. doi: 10.1111/tan.12352 24724906

[23] Ribeiro ÁL Araújo FP Martins JP Loureço AA Huang J Jordana G. A chimeric HLA-A2:β2M:Ig fusion protein for the study of virus-specific CD8^+^ T-cells. J Immunol Methods (2021) 492:1–8:112997. doi: 10.1016/j.jim.2021.112997 33600818

[24] Edgar RC. MUSCLE: multiple sequence alignment with high accuracy and high throughput. Nucleic Acids Res (2004) 32(5):1792–7. doi: 10.1093/nar/gkh340 PMC390337 15034147

[25] Okonechnikov K Golosova O Fursov M UGENE team. Unipro UGENE: a unified bioinformatics toolkit. Bioinformatics (2012) 28(8):1166–7. doi: 10.1093/bioinformatics/bts091 22368248

[26] Drummond AJ Ashton B Buxton S Cheung M Cooper A Wilson A. Geneious v5.5 (2011). Available at: http://www.geneious.com.

[27] Needleman SB Wunsch CD. A general method applicable to the search for similarities in the amino acid sequence of two proteins. J Mol Biol (1970) 48(3):443–53. doi: 10.1016/0022-2836(70)90057-4 5420325

[28] Kyte J Doolittle RF. A simple method for displaying the hydropathic character of a protein. J Mol Biol (1982) 157(1):105–32. doi: 10.1016/0022-2836(82)90515-0 7108955

[29] Smith TF Waterman MS. Identification of common molecular subsequences. J Mol Biol (1981) 147(1):195–7. doi: 10.1016/0022-2836(81)90087-5 7265238

[30] Shimojo N Maloy WL Anderson RW Biddison WE Coligan JE. Specificity of peptide binding by the HLA-A2.1 molecule. J Immunol (1989) 143(9):2939–47. doi: 10.1002/prot.10154 2553813

[31] Doytchinova IA Flower DR. Physicochemical explanation of peptide binding to HLA-A*0201 major histocompatibility complex: a three-dimensional quantitative structure-activity relationship study. Proteins (2002) 48(3):505–18. doi: 10.1002/prot.10154 12112675

[32] Chowell D Krishna S Becker PD Cocita C Shu J Anderson KS. TCR contact residue hydrophobicity is a hallmark of immunogenic CD8+ T cell epitopes. Proc Natl Acad Sci USA (2015) 12(14):E1754–62. doi: 10.1073/pnas.1500973112 PMC4394253 25831525

[33] Smithburn KC Durieux C Koerber R Penna HA Dick GWA Courtois G. International regulation of yellow fever vaccination. In: Smithburn KC Durieux C Koerber R Penna HA Dick GWA Courtois G, editors. Yellow fever vaccination, vol. Annex 2. Geneva: World Health Organization (1956). p. 205–8.

[34] dos Santos CN Post PR Carvalho R Ferreira II Rice CM Galler R. Complete nucleotide sequence of yellow fever virus vaccine strains 17DD and 17D-213. Virus Res (1995) 35(1):35–41. doi: 10.1016/0168-1702(94)00076-o 7754673

[35] Silva NIO Sacchetto L de Rezende IM Trindade GS LaBeaud AD Drumond BP. Recent sylvatic yellow fever virus transmission in Brazil: the news from an old disease. Virol J (2020) 17(1):1–12. doi: 10.1186/s12985-019-1277-7 31973727 PMC6979359

[36] Ritmahan W Kesmir C Vroomans RMA. Revealing factors determining immunodominant responses against dominant epitopes. Immunogenetics (2020) 72(1-2):109–18. doi: 10.1007/s00251-019-01134-9 PMC6971151 31811313

[37] Lázaro S Gamarra D Del Val M. Proteolytic enzymes involved in MHC class I antigen processing: A guerrilla army that partners with the proteasome. Mol Immunol (2015) 68:72–6. doi: 10.1016/j.molimm.2015.04.014 26006050

[38] Falk K Rötzschke O Stevanović S Jung G Rammensee HG. Allele-specific motifs revealed by sequencing of self-peptides eluted from MHC molecules. Nature (1991) 351(6324):290–6. doi: 10.1038/351290a0 1709722

[39] Fruci D Rovero P Falasca G Chersi A Sorrentino R Tosi R. Anchor residue motifs of HLA class-i-binding peptides analyzed by the direct binding of synthetic peptides to HLA class I alpha chains. Hum Immunol (1993) 38(3):187–92. doi: 10.1016/0198-8859(93)90539-d 8106276

[40] Calis JJ Maybeno M Greenbaum JA Weiskopf D De Silva AD Peters B. Properties of MHC class I presented peptides that enhance immunogenicity. PloS Comput Biol (2013) 9(10):1–13:e1003266. doi: 10.1371/journal.pcbi.1003266 PMC3808449 24204222

[41] Rossjohn J Gras S Miles JJ Turner SJ Godfrey DI McCluskey J. T Cell antigen receptor recognition of antigen-presenting molecules. Annu Rev Immunol (2015) 33:169–200. doi: 10.1146/annurev-immunol-032414-112334 25493333

[42] Halagan M Oliveira DC Maiers M Fabreti-Oliveira RA Moraes MEH Porto LC. The distribution of HLA haplotypes in the ethnic groups that make up the Brazilian bone marrow volunteer donor registry (REDOME). Immunogenetics (2018) 70(8):511–22. doi: 10.1007/s00251-018-1059-1 29696367

[43] Ayo CM da Silveira Camargo AV Xavier DH Batista MF Carneiro OA de Mattos LC. Frequencies of allele groups HLA-a, HLA-b and HLA-DRB1 in a population from the northwestern region of são paulo state, Brazil. Int J Immunogenet (2015) 42(1):19–25. doi: 10.1111/iji.12159 25418108

[44] Lund O Nascimento EJ Maciel M Jr Nielsen M Larsen MV Marques ET Jr. Human leukocyte antigen (HLA) class I restricted epitope discovery in yellow fewer and dengue viruses: importance of HLA binding strength. PloS One (2011) 6(10):1–7:e26494. doi: 10.1371/journal.pone.0026494 PMC3198402 22039500

[45] Cleton N Koopmans M Reimerink J Godeke GJ Reusken C. Come fly with me: review of clinically important arboviruses for global travelers. J Clin Virol (2012) 55(3):191–203. doi: 10.1016/j.jcv.2012.07.004 22840968

[46] Klatt MG Mack KN Bai Y Aretz ZEH Nathan LI Scheinberg DA. Solving an MHC allele-specific bias in the reported immunopeptidome. JCI Insight (2020) 5(19):1–11:e141264. doi: 10.1172/jci.insight.141264 PMC7566711 32897882

[47] Akondy RS Monson ND Miller JD Edupuganti S Teuwen D Ahmed R. The yellow fever virus vaccine induces a broad and polyfunctional human memory CD8+ T cell response. J Immunol (2009) 183(12):7919–30. doi: 10.4049/jimmunol.0803903 PMC3374958 19933869

[48] de Melo AB Nascimento EJ Braga-Neto U Dhalia R Silva AM Marques ET. T-Cell memory responses elicited by yellow fever vaccine are targeted to overlapping epitopes containing multiple HLA-I and -II binding motifs. PloS Negl Trop Dis (2013) 7(1):1–11:e1938. doi: 10.1371/journal.pntd.0001938 PMC3561163 23383350

[49] Blom K Braun M Ivarsson MA Gonzalez VD Falconer K Sandberg JK. Temporal dynamics of the primary human T cell response to yellow fever virus 17D as it matures from an effector- to a memory-type response. J Immunol (2013) 190(5):2150–8. doi: 10.4049/jimmunol.1202234 23338234

[50] Fuertes Marraco SA Soneson C Cagnon L Gannon PO Allard M Speiser DE. Long-lasting stem cell-like memory CD8^+^ T cells with a naïve-like profile upon yellow fever vaccination. Sci Transl Med (2015) 7(282):1–15. doi: 10.1126/scitranslmed.aaa3700 25855494

[51] Wieten RW Jonker EF van Leeuwen EM Remmerswaal EB Ten Berge IJ de Bree GJ. A single 17D yellow fever vaccination provides lifelong immunity; characterization of yellow-Fever-Specific neutralizing antibody and T-cell responses after vaccination. PloS One (2016) 11(3):e0149871. doi: 10.1371/journal.pone.0149871 26977808 PMC4792480

[52] Lang A Nikolich-Zugich J. Functional CD8 T cell memory responding to persistent latent infection is maintained for life. J Immunol (2011) 187(7):3759–68. doi: 10.4049/jimmunol.1100666 PMC4102748 21890658

[53] Oberhardt V Luxenburger H Kemming J Schulien I Ciminski K Hofmann M. Rapid and stable mobilization of CD8^+^ T cells by SARS-CoV-2 mRNA vaccine. Nature (2021) 597(7875):268–73. doi: 10.1038/s41586-021-03841-4 PMC8426185 34320609

[54] Martins MA Silva ML Marciano AP Peruhype-Magalhães V Eloi-Santos SM Martins-Filho AO. Activation/modulation of adaptive immunity emerges simultaneously after 17DD yellow fever first-time vaccination: is this the key to prevent severe adverse reactions following immunization? Clin Exp Immunol (2007) 148(1):90–100. doi: 10.1111/j.1365-2249.2006.03317.x 17309541 PMC1868854

[55] Leung CS. Endogenous antigen presentation of MHC class II epitopes through non-autophagic pathways. Front Immunol (2015) 6:464. doi: 10.3389/fimmu.2015.00464 26441969 PMC4563256

[56] Murugesan K Jagannathan P Pham TD Pandey S Bonilla HF Banaei N. Interferon-γ release assay for accurate detection of severe acute respiratory syndrome coronavirus 2 T-cell response. Clin Infect Dis (2021) 73(9):3130–2. doi: 10.1093/cid/ciaa1537 PMC7665338 33035306

[57] Höttler A März L Lübke M Rammensee HG Stevanović S. Broad and efficient activation of memory CD4^+^ T cells by novel HAdV- and HCMV-derived peptide pools. Front Immunol (2021) 12:700438. doi: 10.3389/fimmu.2021.700438 34322126 PMC8312486

[58] Curtsinger JM Agarwal P Lins DC Mescher MF. Autocrine IFN-γ promotes naive CD8 T cell differentiation and synergizes with IFN-α to stimulate strong function. J Immunol (2012) 189(2):659–68. doi: 10.4049/jimmunol.1102727 PMC3392455 22706089

[59] Bhat P Leggatt G Waterhouse N Frazer IH. Interferon-γ derived from cytotoxic lymphocytes directly enhances their motility and cytotoxicity. Cell Death Dis (2017) 8(6):e2836. doi: 10.1038/cddis.2017.67 28569770 PMC5520949

[60] Pan J Zhang M Wang J Wang Q Xia D Cao X. Interferon-gamma is an autocrine mediator for dendritic cell maturation. Immunol Lett (2004) 94(1-2):141–51. doi: 10.1016/j.imlet.2004.05.003 15234546

[61] Frasca L Nasso M Spensieri F Fedele G Palazzo R Ausiello CM. IFN-gamma arms human dendritic cells to perform multiple effector functions. J Immunol (2008) 180(3):1471–81. doi: 10.4049/jimmunol.180.3.1471 18209042

[62] Quintilio W de Freitas FA Rodriguez D Kubrusly FS Yourtov D Raw I. Vitamins as influenza vaccine adjuvant components. Arch Virol (2016) 161(10):2787–95. doi: 10.1007/s00705-016-2994-5 27449155

[63] Karapetyan AR Chaipan C Winkelbach K Wimberger S Jeong JS Seibert V. TCR fingerprinting and off-target peptide identification. Front Immunol (2019) 10:2501. doi: 10.3389/fimmu.2019.02501 31695703 PMC6817589

