# Supplementary figures and images for: In silico and in vitro arboviral MHC class I-restricted-epitope signatures reveal immunodominance and poor overlapping patterns
Source: Front Immunol. 2022 Nov 17;13:1035515. doi: 10.3389/fimmu.2022.1035515 (PMC9713826; doi:10.3389/fimmu.2022.1035515)

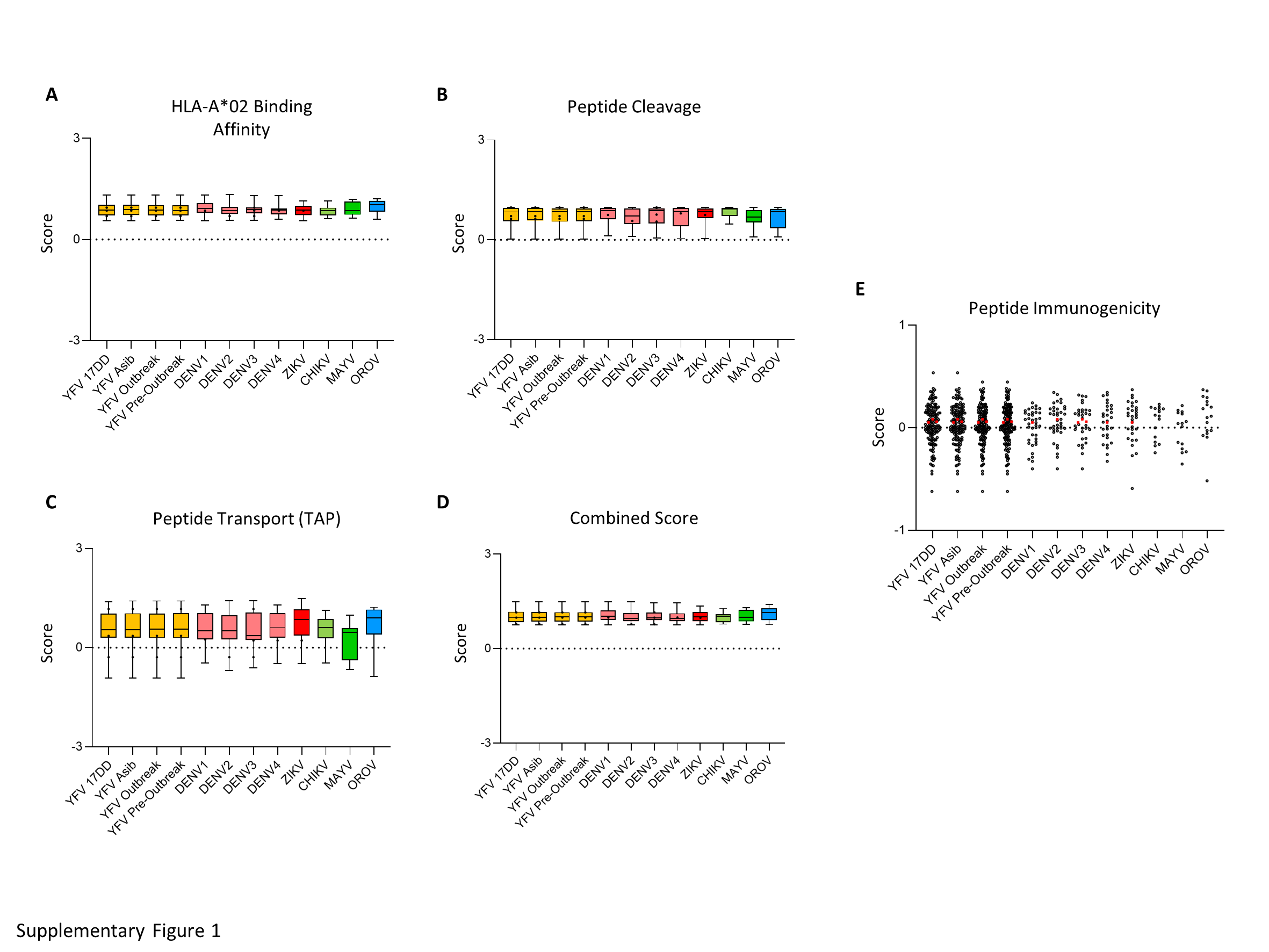

Supplement: Supplementary file 1 [file Image_1.tif]

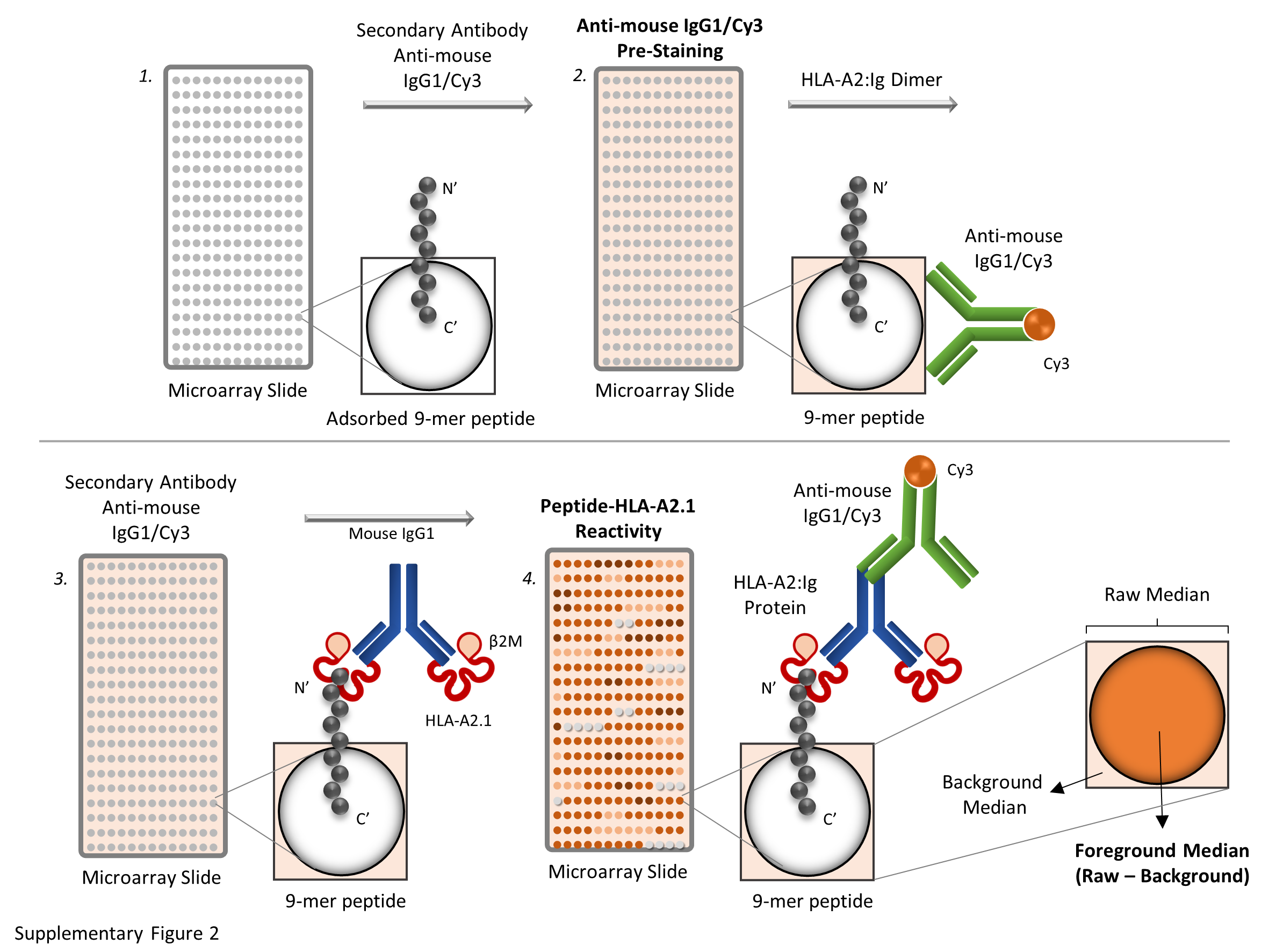

Supplement: Supplementary file 2 [file Image_2.tif]

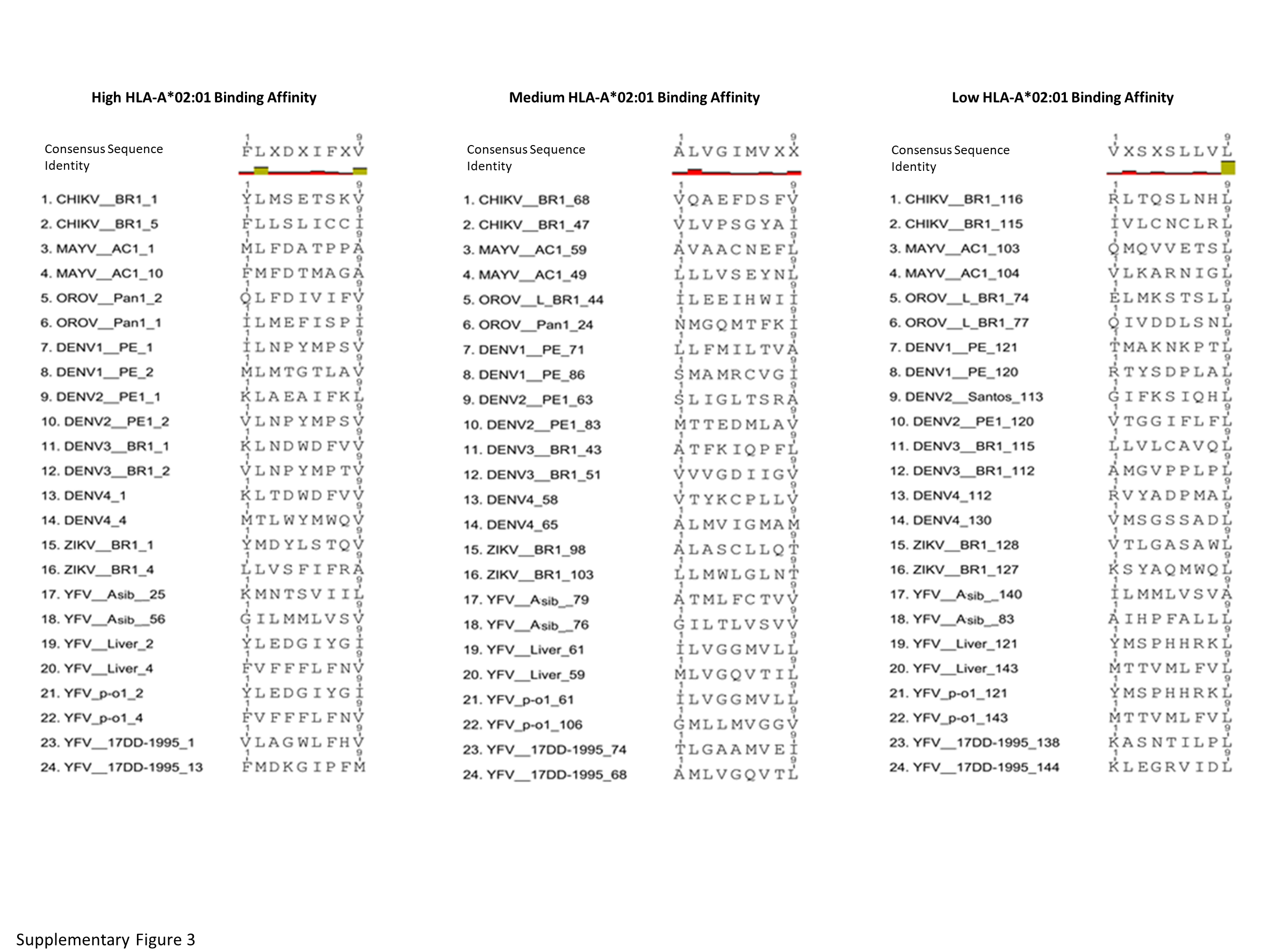

Supplement: Supplementary file 3 [file Image_3.tif]
